# Prevalence of Alcohol Use and Associated Factors among Dilla University Students, Dilla Town, Southern Ethiopia: A Cross-Sectional Study

**DOI:** 10.1155/2020/3971090

**Published:** 2020-10-18

**Authors:** Yigrem Ali Chekole

**Affiliations:** Department of Psychiatry, College of Medicine and Health Sciences, Dilla University, Dilla, Ethiopia

## Abstract

**Introduction:**

Globally, alcohol is one of the most prevalent forms of substance use that is particularly high among young age groups. Despite the adverse health and social challenges associated with alcohol use, it is one of the most common risky behaviours among university students.

**Objective:**

This study aimed to assess the prevalence of alcohol use and associated factors among Dilla University students in Southern Ethiopia.

**Methods:**

An institution-based cross-sectional study was conducted at Dilla University among undergraduate regular students from January to February 2018. A systematic random sampling technique was used to get a total sample of 803 students each year from the department of the university. The collected data were coded, entered into Epi Info version 7.1, and analyzed with SPSS version 20.

**Results:**

A total of 803 participants were successfully interviewed with a response rate of 91.7%. Alcohol use prevalence was 41.8% (*n* = 336) among participants. Being in fourth year (AOR = 2.66, 95% CI: 1.64, 4.31), having friends who use the substance (AOR = 1.53, 95% CI: 1.09, 2.1), being a khat user (AOR = 1.48, 95% CI: 1.05, 2.09), and being a cigarette smoker (AOR = 1.76, 95% CI: 1.09, 2.84) were found to be significantly associated with alcohol use.

**Conclusion:**

The current study revealed that fourth-year students, having friends who use the substance, being khat user, and being cigarette smoker had higher odds of alcohol use among the students. So the findings suggest that effective campus-based counselling and peer education should be implemented for early prevention, detection, and alleviation of alcohol use among students in the university.

## 1. Introduction

Substance use among adolescents can lead to a variety of detrimental consequences. Cigarette smoking, alcohol drinking, and cannabis use can increase accidental or intentional injuries, commission of crimes, mood disorders, and mortality and can complicate normal psychosocial development [1–3].

The use of substances is one of the rising major public health and socioeconomic problems worldwide, which has dramatically increased particularly in developing countries [4–8]. Substance use in Africa is increasing rapidly, especially for tobacco, alcohol, and khat [9].

Alcohol is one psychoactive substance that has been widely used in many cultures for centuries [10, 11]. It is often consumed before, along with, or after other psychoactive substances, and the comorbidity of alcohol and tobacco dependence is strong [12]. Alcohol use causes large disease and social and economic burden in many societies [13].

Harmful use of alcohol accounts for nearly 6.5% of all deaths in Europe, where alcohol-related harm is highest, and even in the world, but the burden is much higher in certain countries and for certain groups within countries [14]. About 3.3 million deaths, or 5.9% of all global deaths, were attributable to alcohol use [11, 13]. There are significant gender differences in the proportion of global deaths attributable to alcohol, for example, 7.6% of deaths among males and 4.0% of deaths among females were attributable to alcohol [13].

Younger people are more affected by alcohol than older people. Possibly 13.5% of all deaths among those who are 20–39 years of age may be attributed to alcohol [12]. An estimated 24.6 million Americans aged 12 or older had used an illegal drug, approximately 9.4% of the population [15]. In most parts of Africa, health and social problems related to alcohol use are increasing. In sub-Saharan Africa, an estimated 1.8% of the disease burden is attributable to alcohol use [16, 17].

In a study done in western Kenya among college students, the lifetime prevalence rate of alcohol use was 51.9% [18]. Being male, ease of getting an adult to buy alcohol, cigarette use, and having multiple sexual partners were risk factors [11].

About 5.9% of all global deaths were attributable to alcohol consumption. Global deaths were attributable to alcohol among males (7.6%) and females (4.0%) [14].

A study done in western Kenya among college students showed that 75.1% were introduced to substance use by a friend while 23.5% were introduced by a relative. Majority of users wanted to relax (62.2%) or relieve stress (60.8%) [18].

Over 20% of out-of-school youth had unprotected sex during the 12 months before the interview compared to 1.4% of in-school youth. Daily alcohol intake and khat intake were also associated with unprotected sex [19].

One Ethiopian study of Haramaya University students found the prevalence of substance use was high, and about two-thirds (62.4%) of the participants used at least one substance, most commonly alcohol (50.2%) [20].

Another study at Axum University found that the lifetime prevalence of alcohol drinking was 34.5% and the current prevalence was 32.8% [21]. Similarly, in Debre Markos University, the prevalence of alcohol drinking was 33.8% [22].

Another study done in Hawassa found that 40.8% used alcohol, 20.3% chewed khat, 11.9% smoked cigarettes, and 0.9% used marijuana [23]. In another study among RVUC students, the lifetime prevalence of alcohol drinking was 40.2%. Similarly, the current prevalence of alcohol drinking was 35.6% [24].

The Hawassa study found that having family members who used psychoactive substances (PAS), peer influence, being male, and living alone during school age were positively associated with overall PAS use in the past 12 months [23]. The most common reasons for use among university students were for relaxation with friends (84 (53.8%)), peer pressure (72 (46.2%)), and to get relief from stress (56 (35.9%)), respectively [23].

As in many developing countries, alcohol is the most frequently used substance in Ethiopia [6, 25].

Therefore, the present study aimed at assessing the prevalence of alcohol use and associated factors among Dilla University students. The findings are expected to inform university students about the possible dangers of alcohol use and to contribute significantly to national guidelines, targets, policies, and strategies that pertain to the management and evaluation of youth health programs in universities.

## 2. Methods and Materials

### 2.1. Study Design, Period, and Study Area

The institutional-based cross-sectional study was conducted from January to February 2018 among Dilla University students in the Gedeo Zone, Southern Ethiopia, which is found on the main road from Addis Ababa to Kenya 360 km south of Addis Ababa and 90 km south of Hawassa (capital of SNNPR). The Gedeo Zone has a longitude and latitude of 6°24′30″N 38°18′30″E with an elevation of 1570 meters above sea level [26, 27].

Dilla University is one of the public universities located in the fertile coffee belt of the Gedeo Zone. Its history can be traced back to the year 1996 when it was called Dilla College of Teacher Education and Health Sciences and was included in the then newly emerging University, Debub University in 2001 EC, and became Dilla University in 2004 EC. It has three campuses, six colleges, three schools, and two institutes offering undergraduate and postgraduate programmes. Currently, more than 31,000 students take part in regular, summer, weekend, and distance programmes.

### 2.2. Sample Size and Sampling Technique

The sample size was determined using a single population proportion formula by an assumption of *p*= 45.7%, based on a previous study from Southern Ethiopia (Hawassa University) [23] with 95% confidence interval and 5% margin of error. Assuming a 15% nonresponse rate and a design effect of two for multistage sampling, the total sample size was computed to be 876.

All regular undergraduate students attending at Dilla University were eligible to participate. A multistage sampling technique was used to select the study participants. First, students were divided into their colleges and departments. Then, further stratification was used based on the year of study. Students from each year of study were allocated proportionally to the class size of their departments. “*K*” was calculated as 12138 ÷ 876 = 13.86∼14 for each year separately. Therefore, the study subjects were selected every 14 from the list of students' names.

### 2.3. Data Collection Procedures and Instruments

A structured questionnaire adapted from the previous literature on alcohol use was used to collect the data. The questionnaire had different components including sociodemographic, clinical, and alcohol use characteristics. Sociodemographic characteristics included age, sex, religion, ethnicity, monthly income, place of residency, types of high school attended, current living, year of study, friends' psychoactive substance use, and family's psychoactive substance use.

The clinical factors and psychoactive substances included the diagnosis of mental illness, family history of mental illness, history of chronic illness, khat use, and cigarette smoking.

Alcohol use was measured by a 9-item semistructured questionnaire which was prepared by reviewing the WHO students' drug use survey questionnaire, the ASSIST guideline [28], and the relevant previous literature [6, 8, 18, 22, 24, 29–32]. Alcohol, Smoking, and Substance Involvement Screening Test (ASSIST) guideline specifically asked about the following: (1) all substances a participant had ever used in his/her lifetime, (2) frequency of use in the past three months preceding the survey, (3) frequency of experiencing a strong desire to use a substance in the past three months preceding the survey, (4) frequency of health, social, legal, or economic problems related to substance use in the past three months preceding the survey, (5) frequency of substance use interfering with students' roles and responsibilities in the past three months preceding the survey, (6) if she/he has a friend or a relative or anyone else ever expressed concern about your use of the substance, and (7) if she/he has ever tried and failed to control, cut down, or stop using the substance. The questions are in increasing order of specificity. The questionnaire explicitly asked questions about alcohol, cigarette, khat, and cannabis use as these are recognized as the most frequently tried from other studies [28]. Participants indicated their responses to the questionnaire with pen and paper.

### 2.4. Measurement

The outcome variable (dependent variable) was “alcohol use” among Dilla University students. Thus, the independent variables used for multiple logistic regression analysis were age, sex, religion, ethnicity, monthly income, place of residency, types of high school attended, current living, year of study, friends' psychoactive substance use, family's psychoactive substance use, diagnosis of mental illness, family history of mental illness, history of chronic illness, khat use, and cigarette smoking.

### 2.5. Data Quality Assurance

The pretest was done on students with 5% of the sample size in Hawassa University. Data collectors and supervisors were trained on the data collection tool and sampling techniques. Supervision was held regularly during the data collection period by both the researcher and supervisors. The wholeness and consistency of the data were crosschecked and cleaned manually.

### 2.6. Statistical Analysis and Processing

Data were entered into Epi Info version 7 computer programs and transported to SPSS version 20 computer program for further analysis and cleaning. Descriptive statistics were used to determine alcohol use and associated factors of participants. Tables were used for the data presentation. Associations of alcohol use variables and demographic characteristics were analyzed using the chi-square test, Fisher's exact test, and binary logistic regression with odds ratio and 95% CI.

Multivariate logistic regression analysis was carried out to examine the associations between each independent variable and the outcome variable. The model was checked for fitness with *R*-squared value. An *R*-squared value greater than 50% was considered as good. Hosmer and Lemeshow goodness-of-fit test was also used to check the model fitness. All variables with a *p* value of ≤0.25 in the bivariable analysis were considered as the candidate for multivariable regression to control possible confounders. Finally, variables with a *p* value of <0.05 were having a statistically significant association with alcohol use at corresponding 95% CI.

### 2.7. Ethical Clearance

Ethical approval was obtained from the Institutional Review Board of Dilla University. The purpose and importance of the study were explained to each participant before they proceed into actual activities. Confidentiality was maintained by anonymous questionnaires, and informed consent was obtained from each participant.

## 3. Results

A total of 876 participants were recruited, and 803 participants completed the questionnaires sufficiently for analysis, giving the overall response rate of 91.7%.

### 3.1. Sociodemographic Characteristics

Most of the study subjects were in the age group of 20–24 years (81.7%; *n* = 656), and most were male (66.9%; *n* = 537), single (95.0%; *n* = 763), and Ethiopian Orthodox Tewahedo Christians (58.4%; *n* = 469). Over half of the participants lived rurally before the university (57.2%; *n* = 459), and almost all lived currently in halls of residence (99.0%; *n* = 795). Most had attended a public high school (88.9%; *n* = 714). (Table 1).

### 3.2. Clinical Characteristics

Of the respondents, 6.4% (*n* = 51) reported a history of mental illness while 13.8% (*n* = 111) reported a family history of mental illness and 14.3% (*n* = 115) had a chronic physical illness.

### 3.3. Habit of Alcohol Drinking in Respondents

Participants who have a habit of alcohol use were 41.8% (*n* = 336) and with current use in the last 30 days were 24.4% (*n* = 196). The most common reasons for alcohol use were for pleasure and to socialize (18.7% (*n* = 150) and 10.7% (*n* = 86), respectively).

Participants' main reasons for avoiding alcohol were for the religious and economic reasons (19.3% (*n* = 155) and 18.7% (*n* = 150), respectively). However, the study participants' initiation of alcohol use was by friends and family (19.1% (*n* = 153) and 8.7% (*n* = 70), respectively) (Table 2).

### 3.4. Factors Associated with Alcohol Use among Students

In bivariate analyses, age, sex, religion, ethnicity, year of study, friends' use of psychoactive substances, family's psychoactive substance use, diagnosis of mental illness, family history of mental illness, history of chronic illness, khat use, and cigarette smoking were analyzed.

Multivariate logistic regression was also used to analyze associations between variables which have a *p* value of <0.2 in bivariate logistic regression. After adjusting for possible covariates, year of study, being friends with psychoactive substance user, khat use, and cigarette smoking were significantly associated with alcohol use among students with a *p* value<0.05 and with *R*-squared value of 0.573 (57.30 in percent).

Fourth-year students were about 2.66 times more likely to have alcohol use as compared to first-year students (AOR = 2.66, 95% CI: 1.64, 4.31).

Students having friends who were psychoactive substance users were 1.53 times more likely to use alcohol on the campus as compared with others (AOR = 1.53, 95% CI: 1.09, 2.1).

Those who had used khat were about 1.48 times more likely to have used alcohol compared to nonkhat users (AOR = 1.48, 95% CI: 1.05, 2.09). Those who smoked cigarettes were about 1.76 times more likely to use alcohol compared to nonsmokers (AOR = 1.76, 95% CI: 1.09, 2.84) (Table 3).

## 4. Discussion

The study has tried to determine the prevalence of alcohol use among 803 students and associated factors using a cross-sectional study design and a semistructured questionnaire. The study participants were predominantly males (66.9%; *n* = 537), which is similar to previous studies [11, 18]. Of the participants, 41.8% were lifetime users of alcohol, which is comparable to other Ethiopian student surveys (40.8% in [23] and 40.2% in [24]). Alcohol prevalence was significantly lower than a similar study done in Kenya (51.9%) [18] and Ethiopia (50.2%) [29]. However, prevalence here was significantly higher than a similar study done in Axum University (34.5%) [6] and Debre Markos University (33.8%) in Ethiopia [22]. The possible explanation for the observed difference could be due to the difference in alcohol accessibility, knowledge, and the study area.

The prevalence of current alcohol use in the last 30 days was 24.4%. The finding was in line with a study done in Addis Ababa University among medical students (22%) (25% males vs. 14% females) [6], which is lower compared to a study done among Rift Valley University College students (35.6%) in Ethiopia [24]. The possible reason might be the university setup and knowledge of the students.

The main reasons for alcohol use given by participants were for pleasure and to socialize (18.7% (*n* = 150) and 10.7% (*n* = 86), respectively). Similar studies done in Western Kenya among college students reported 62.2% or 60.8% to relax or relieve stress, respectively [18], a study in Ethiopia, among Debre Markos University students, reported 79.0% for relaxation [22], and a study among Axum University students reported 65.5% for relaxation or 37.7% to relieve stress, respectively [21]. Also, a large proportion of the study participants were introduced to alcohol use by friends and family (19.1% (*n* = 153) and 70 (8.7% (*n* = 70), respectively), which is similar to studies done in Western Kenya among college students showing 75.1% by friends and 23.5% by relatives [18] and a study among Debre Markos Universtiy students is showing that 63% of students were introduced to alcohol use by friends in Ethiopia [22].

Here, alcohol use prevalence increased with the seniority of the students. The fourth-year students were about 3 times more likely to have alcohol use as compared to first-year students. Similar findings were reported in Hawassa and Haramaya Universities of Ethiopia [20, 23], Sudan [33], and Nigeria [34].

Students having friends who use psychoactive substances were 1.53 times more likely to use alcohol on the campuses as compared with their counterparts. The study is in line with a study done in Axum University among students; having family members and peer friends who drink alcohol were strongly associated with alcohol use [21]. Also, the finding was similar to a study done in Ethiopia among students whose friends consume alcohol were more likely to consume alcohol [35].

In this study, khat chewers had a strong association with alcohol use being about 1.48 times more likely to use alcohol as compared to counterparts. This study supported by studies of emergency hospital cases has found that a wide variety of controlled psychoactive substances are commonly taken before, along with, or after alcohol, and both substances are affecting the body [15]. The reason might require joint effect or augment to diminish or counteract the effect of the first substance.

Cigarette smoking had a strong positive association with alcohol use with smokers being twice as likely to use alcohol compared to their counterparts. Similarly, a study done in Ethiopia among students showed that the use of cigarette was also significantly associated with alcohol consumption [21, 36, 37]; the two tend to co-occur. The other common reason is that many cigarette smokers use alcohol to reduce the effect of smoking.

Chewing khat was also associated with alcohol use, with chewers being 1.48 times more likely to use alcohol than nonchewers. Studies of emergency hospital cases have also found that a wide variety of psychoactive substances are commonly taken before, along with, or after alcohol [15].

However, in contrast to other studies, here sex, age, family history of substance use, and student's origin of residence were not associated with alcohol use [6, 37, 38].

## 5. Conclusion

In conclusion, the prevalence of alcohol use among students at Dilla University was high. Being a 4th-year student, having friends who use substances, being khat user, and being cigarette smoker were associated significantly with alcohol use. In Ethiopia, being a university student appears to increase alcohol use. The findings suggest that effective campus-based counselling and peer education should be implemented for early prevention, detection, and alleviation of alcohol use among students. Moreover, longitudinal studies should be showed to check the reproducibility of alcohol use among university students in Ethiopia.

## Figures and Tables

**Table 1:** Sociodemographic characteristics of respondents at Dilla University, SNNPR, Ethiopia, 2018.

Variables	Categories	Frequency	Percentage (%)
Sex	Male	537	66.9
	Female	266	33.1

Age	15–19	78	9.7
	20–24	656	81.7
	25–30	69	8.6

Marital status	Married	37	4.6
	Single	763	95.0
	Divorced/widowed	3	0.4

Religion	Orthodox	469	58.4
	Muslim	134	16.7
	Protestant	171	21.3
	Others^*∗*^	29	3.6

Ethnicity	Oromo	305	38.0
	Amhara	289	36.0
	Gurage	61	7.6
	Tigray	114	14.2
	Others^*∗∗*^	34	4.2

Place of residence	Urban	344	42.8
	Rural	459	57.2

Monthly income	<100	49	6.1
	101–299	175	21.8
	300–499	285	35.5
	>500	294	36.6

Current life	Dormitory	795	99.0
	Nondormitory	8	1.0

Type of high school attended	Public high school	714	88.9
	Private high school	66	8.2
	Missionary high school	23	2.9

Year of study	1^st^ year	242	30.1
	2^nd^ year	232	28.9
	3^rd^ year	144	17.9
	4^th^ year	126	15.7
	5^th^ year	59	7.3

^*∗*^Catholic, Jehovah's witnesses, and no religion; ^*∗∗*^Gedeo, Wolayta, Siltie, and Sidama.

**Table 2:** Frequency and percentage of alcohol drinking of respondents in Dilla University, SNNPR, Ethiopia, 2018.

Variables	Categories	Frequency (%)	Percentage
Alcohol use	Yes	336	41.8
	No	467	58.2

Recurrence of alcohol use	Every day	26	3.2
	Once per week	60	7.5
	2–3 times per week	33	4.1
	Current use in the last 30 days	196	24.4
	Others	21	2.6

Length of alcohol use	6 months	48	6
	1 year	68	8.5
	2 years	86	10.7
	>2 years	134	16.7

Reasons for alcohol use	For pleasure	150	18.7
	For academic purpose	33	4.1
	To socialize	86	10.7
	To prevent withdrawal effect	11	1.4
	To pass time	43	5.4
	Others	13	1.6

Reasons for not using alcohol	Religious reason	155	19.3
	Health reason	69	8.6
	Economic	150	18.7
	Lack of desire	43	5.4
	Others	49	6.1

Intention for alcohol use	Yes	44	5.5
	No	422	52.6

Initiation of alcohol use	Friends	153	19.1
	Religious custom	26	3.2
	Accidental	62	7.7
	Family	70	8.7
	Traditional healers	15	1.9
	Others	9	1.1

Need for stopping alcohol use	Yes	94	11.7
	No	241	30.0

Potential reasons for stopping alcohol use	If I found that alcohol is a cause for my health problem	224	27.9
	Immediately after graduation	47	5.9
	If I have a financial problem	29	3.6
	If my beloved is against it	23	2.9
	Others	12	1.5

**Table 3:** Bivariate and multivariate logistic regression analysis showing alcohol drinking among Dilla University students in Ethiopia, 2018.

Variables	Categories	Alcohol use		COR (95% CI)	AOR (95% CI)
		Yes	No		
Age	15–19	26	52	1	1
	20–24	276	380	1.45 (0.89, 2.39)	1.25 (0.73, 2.15)
	25–30	34	35	1.94 (1.00, 3.74)	1.01 (0.47, 2.20)

Sex	Male	236	301	1.30 (0.96, 1.75)	0.93 (0.66, 1.31)
	Female	100	166	1	1

Year of study	1^st^ year	90	152	1	1
	2^st^ year	79	153	0.87 (0.6, 1.27)	0.75 (0.50, 1.12)
	3^rd^ year	60	84	1.21 (0.79, 1.84)	0.98 (0.62, 1.55)
	4^th^ year	77	49	2.65 (1.7, 4.13)	2.66 (1.64, 4.31)^*∗∗∗*^
	5^th^ year	30	29	1.75 (0.99, 3.10)	1.47 (0.77, 2.81)

Friends' PAS use	Yes	133	121	1.87 (1.39, 2.53)	1.53 (1.09, 2.1)^*∗*^
	No	203	346	1	1

Family's PAS use	Yes	91	94	1.47 (1.06, 2.05)	1.29 (0.89, 1.86)
	No	245	373	1	1

Khat use	Yes	153	157	1.559 (1.17, 2.07)	1.48 (1.05, 2.09)^*∗*^
	No	183	310	1	—

Cigarette use	Yes	59	41	2.21 (1.45, 3.39)	1.76 (1.09, 2.84)^*∗*^
	No	277	426	1	1

^*∗*^ *p* value is significant at *p* < 0.05.^*∗∗∗*^*p* value is significant at *p* < 0.001. PAS = psychoactive substance.

## Data Availability

The data used to support the finding of this study are available from the author upon request.

## Abbreviations

ASSIST: Alcohol, Smoking, and Substance Involvement Screening Test
DU: Dilla University
Epi Info: Epidemiological Information
PAS: Psychoactive substance
RVUC: Refit Valley University College
SNNPR: Southern Nations, Nationalities, and People's Region
SPSS: Statistical Package for Social Science
WHO: World Health Organization.

## Additional Points

This study did not do a detailed validation of the alcohol screening tool. The collected data were measured using a self-reported questionnaire, but it was safeguarded anonymity to reduce subjectivity bias. The cross-sectional nature of the study design might not show the causal relationships between sociodemographic and risk factors and alcohol use.t
